# Congenital Dermatofibrosarcoma Protuberans—An Update on the Ongoing Diagnostic Challenges

**DOI:** 10.3390/cancers17010158

**Published:** 2025-01-06

**Authors:** Fortunato Cassalia, Andrea Danese, Enrico Cocchi, Silvia Vaienti, Anna Bolzon, Ludovica Franceschin, Roberto Mazzetto, Francesca Caroppo, Davide Melandri, Anna Belloni Fortina

**Affiliations:** 1 Unit of Dermatology, Department of Medicine, University of Padova, 35122 Padua, Italy; anna.bolzon.1@studenti.unipd.it (A.B.); ludovica.franceschin@studenti.unipd.it (L.F.); roberto.mazzetto@studenti.unipd.it (R.M.); francesca.caroppo@unipd.it (F.C.); anna.bellonifortina@unipd.it (A.B.F.); 2 Unit of Dermatology, Department of Medicine, University of Verona, 37129 Verona, Italy; andrea.danese_02@studenti.univr.it (A.D.); silvia.vaienti@studenti.univr.it (S.V.); 3 Department of Medical and Surgical Sciences, University of Bologna, 40138 Bologna, Italy; enrico.cocchi3@unibo.it (E.C.); davide.melandri@auslromagna.it (D.M.); 4 Department of Precision Medicine and Genomics, Columbia University, New York, NY 10027, USA; 5 Neonatal and Pediatric Intensive Care Unit, AUSL Romagna, 48121 Ravenna, Italy; 6 Regional Center for Pediatric Dermatology, Department of Women’s and Children’s Health (SDB), University of Padua, 35122 Padua, Italy; 7 European Reference Network for Rare Skin Disorders-ERN Skin, Department of Women and Children’s Health (SDB), University of Padua, 35122 Padua, Italy; 8 Dermatology Unit and Burn Center, AUSL Romagna, Cesena Hospital, 47521 Cesena, Italy

**Keywords:** congenital DFSP, misdiagnosis, pediatric sarcoma, dermatofibrosarcoma protuberans, pediatric skin cancer, rare skin cancer, dermatology, soft tissue tumor

## Abstract

Dermatofibrosarcoma protuberans (DFSP) is a rare low-grade soft tissue sarcoma. Its congenital form poses distinct diagnostic and therapeutic challenges, often resembling benign entities like hemangiomas or vascular malformations, which can lead to misdiagnosis and treatment delays. This review delves into the epidemiology, clinical manifestations, and diagnostic strategies for DFSP, with an emphasis on the use of advanced molecular techniques, such as FISH and RT-PCR, for the detection of the COL1A1-PDGFB fusion gene, a definitive marker of DFSP. We also explore emerging non-invasive imaging technologies, including multispectral and optical coherence, which, while promising, still require further validation. This review underscores the importance of differential diagnosis from similar conditions, such as medallion-like dermal dendritic hamartoma (MLDDH), through an integrated approach that combines histopathology, immunohistochemistry, and molecular testing. Surgical excision, particularly using Mohs micrographic surgery, is preferred to achieve oncologic safety while preserving aesthetic and functional results in pediatric cases. Furthermore, we discuss the potential of adjuvant therapies like tyrosine kinase inhibitors in treating advanced or inoperable cases. Highlighting the critical role of early detection and a collaborative treatment strategy, this review aims to enhance diagnostic precision and access to innovative treatments, ensuring optimal management for this severe, yet rare, condition.

## Simple Summary

Congenital dermatofibrosarcoma protuberans (DFSP) is a rare skin cancer in infants that often mimics benign lesions such as birthmarks or hemangiomas, leading to misdiagnosis and treatment delays. Early biopsy and accurate diagnosis are essential to prevent aggressive growth and recurrence. Multidisciplinary management and clinician awareness are essential to improve outcomes in affected children.

## 1. Introduction

Dermatofibrosarcoma protuberans (DFSP) is a rare, low-grade sarcoma that presents diagnostic challenges due to its resemblance to benign lesions [1]. Congenital DFSP significantly deviates from its adult counterpart in terms of its clinical presentation, growth dynamics, and treatment approaches. These lesions are commonly mistaken for benign conditions such as hemangiomas, vascular malformations, or benign fibrous nevi, leading to frequent misdiagnoses or delays in diagnosis, which can negatively impact treatment outcomes [2,3,4]. Congenital DFSP management requires meticulous care to maintain function and aesthetics in developing children [4]. Achieving complete excision with clear margins is crucial to prevent recurrence, and efforts must be made to minimize disfigurement and functional limitations. The tumor’s indolent progression and its resemblance to benign lesions highlight the necessity for heightened clinical vigilance to facilitate early diagnosis [5,6]. DFSP diagnosis relies on integrated approaches including clinical evaluation, biopsy, histopathology, and the use of advanced tools for molecular characterization [7]. Among these, identifying the COL1A1-PDGFB fusion gene, resulting from the chromosomal translocation t(17;22) (q22;q13), is a key element for diagnostic confirmation. Techniques like fluorescence in situ hybridization (FISH) and reverse transcription–polymerase chain reaction (RT-PCR) are instrumental in detecting the genetic alterations characteristic of DFSP and differentiate it from benign lesions that might exhibit clinical and histopathological overlapping features [8]. A relevant example is the Medallion-like Dermal Dendrocyte Hamartoma (MLDDH), a rare benign lesion that may exhibit spindle cell proliferation and CD34 positivity but differs from DFSP due to its absence of the COL1A1-PDGFB fusion gene and strong positivity for factor XIIIa [8,9,10,11]. The incorporation of imaging techniques into the diagnostic framework offers additional support. Magnetic resonance imaging (MRI) is especially valuable for determining the lesion’s extent and its impact on surrounding soft tissues. Computed tomography (CT) is useful for detecting deeper infiltration into adjacent tissues, including muscles and, in more advanced cases, bones [12,13]. The standard treatment for congenital DFSP is surgical excision, with Mohs micrographic surgery (MMS) being the preferred method. MMS allows for the complete removal of the tumor while conserving healthy tissue, which is crucial for minimizing functional and cosmetic effects in pediatric patients. Alternatively, wide local excision (WLE) with margins of 2–5 cm can be used, but this method poses challenges in neonates due to their smaller anatomical structures and the potential impacts on growth [5,6,7,12]. Emerging therapeutic options, such as tyrosine kinase inhibitors like imatinib mesylate targeting the COL1A1-PDGFB fusion gene, present alternatives in specific cases, especially when surgery is not viable [14]. This narrative review explores the complexities of diagnosing and managing congenital DFSP, emphasizing the importance of clinical vigilance and timely intervention. Early and accurate diagnosis can reduce the need for extensive surgery, improve clinical outcomes, and enhance the quality of life for pediatric patients. The goal of the present work is to enhance clinicians’ ability to recognize this rare cancer and provide comprehensive care through a multidisciplinary team including dermatologists, pediatric surgeons, oncologists, and pathologists.

## 2. Epidemiology

DFSP is a rare, low-grade soft tissue sarcoma that originates from the dermal layer of the skin. Representing less than 0.1% of all malignancies and about 1% of soft tissue sarcomas, DFSP is known for its slow growth and locally invasive nature. Although it typically occurs in adults aged 20 to 50 [2,4,15,16], congenital DFSP accounts for approximately 6% of all DFSP cases, with most lesions presenting at birth or during the first year of life. This rare condition presents diagnostic challenges, often being misdiagnosed as more common benign skin lesions present at birth, such as hemangiomas, vascular malformations, or pigmented birthmarks, leading to delays in appropriate treatment [2,17]. There is no significant gender predilection in congenital DFSP, with studies showing nearly equal representation of males and females [17]. This lack of gender segregation reinforces the need for clinicians to consider congenital DFSP in all pediatric patients presenting with suspicious skin lesions, regardless of gender [17]. The extreme rarity of congenital DFSP means that many clinicians never encounter a case, resulting in diagnostic challenges and oversight [15,16]. This unfamiliarity further underscores the critical importance of raising awareness about congenital DFSP among healthcare providers. Enhanced education and a high index of suspicion are essential for early diagnosis, leading to more effective management strategies, reduced surgical morbidity, and improved long-term outcomes for affected children.

## 3. Key Differences Between Adult and Congenital DFSP

### 3.1. Onset and Presentation

Adult DFSP typically develops between the ages of 20 and 50 and presents as firm, raised nodules or masses. Congenital DFSP appears at birth or within the first year of life, usually as flat or slightly raised plaques, often mimicking benign lesions such as hemangiomas or vascular malformations [2,4,17].

### 3.2. Growth Pattern

In adults, DFSP shows slow, steady growth with the potential for deep infiltration over time. Congenital DFSP, although initially subtle, may show more rapid local growth if not diagnosed early [15,16].

### 3.3. Diagnostic Challenges

Adult DFSPs are often mistaken for benign soft tissue tumors such as lipomas, whereas congenital DFSPs are often misdiagnosed as benign pediatric conditions due to their atypical presentation and similarity to non-aggressive lesions [4,15,16].

### 3.4. Histopathology and Molecular Features

Both forms share the storiform pattern of spindle cells and CD34 positivity. However, molecular confirmation of the COL1A1-PDGFB fusion gene is critical, particularly in congenital cases, to differentiate it from benign mimickers such as medallion-like dermalendrocyte hamartoma (MLDDH) [8,9,10,11].

### 3.5. Therapeutic Considerations

Treatment of adult DFSP focuses on achieving clear margins with wide local excision (WLE) or Mohs micrographic surgery (MMS). In congenital DFSP, the smaller anatomical structures and the need to preserve growth and aesthetics necessitate precise, tissue-sparing approaches, making MMS particularly advantageous [5,6,7,12].

## 4. Clinical Presentations

### 4.1. Lesion Characteristics

DFSP presents a variety of morphological characteristics that often complicate diagnosis. Typically, congenital DFSP appears as a slow-growing, firm plaque or nodule [6]. These lesions can be skin-colored, reddish, purplish, or even bluish, which often leads them to be confused with more common vascular lesions such as hemangiomas. Less frequently, congenital DFSP can present as a mass or a pigmented lesion, further adding to the diagnostic challenges, as these presentations can mimic other benign or even malignant conditions [3,18].

- **Plaques**: Plaques are the most common presentation of congenital DFSP, typically appearing as flat or slightly raised areas that are firm when palpated. They can resemble benign dermatofibromas, keloids, or hypertrophic scars, leading to delays in suspicion and appropriate diagnosis [19,20,21].
- **Nodules**: Nodular forms of DFSP are firm, well-circumscribed masses that may resemble benign tumors such as lipomas, cysts, or neurofibromas. This resemblance often leads to initial misclassification as a benign soft tissue tumor [22,23,24].
- **Masses**: Larger DFSP lesions may present as masses that can be mistaken for more aggressive malignancies or significant benign growths, such as soft tissue sarcomas or deep-seated lipomas [14,22,23].
- **Pigmented Lesions**: Pigmented DFSP is rare but challenging to diagnose due to its similarity to melanocytic nevi, especially in atypical cases. The pigmented variant, also known as Bednar tumors, can further confound clinicians during early evaluation [18,25].

### 4.2. Common Sites

Lesions can occur anywhere on the body, but they are most frequently found on the trunk, followed by the proximal extremities (Table 1). Certain anatomical sites, particularly in congenital presentations, pose a higher risk of misdiagnosis due to the complexity of the differential diagnosis and the overlap with benign conditions [22,23,26,27,28].

- **Head and Neck Region:** The complexity of the head and neck region, with its variety of benign skin structures, increases the risk of misdiagnosis. Congenital DFSP in these locations may be confused with benign cysts, vascular anomalies, or other nodular growths [3,22,26].
- **Lower Limbs:** The lower limbs are a common site for DFSP lesions in children. However, these lesions are often mistaken for benign soft tissue growths, such as dermatofibromas, due to their common occurrence in this area [19,23].
- **Trunk:** The trunk is a frequent site for DFSP and is also a common location for benign conditions like keloids, cysts, or other dermatofibromas, which often leads to misdiagnosis [19,20,27].
- **Genital Area:** The genital area has a lower rate of misdiagnosis, possibly due to more thorough evaluations being conducted because of the anatomical sensitivity. Lesions in this region typically prompt careful examination, which aids in correct identification [4,28].

## 5. Diagnostic Challenges

### 5.1. Misdiagnosis

The diagnosis of congenital DFSP is particularly challenging due to its rarity and its frequent resemblance to benign skin conditions [3,15,16]. As a result, it is often initially misdiagnosed, leading to significant treatment delays and adverse patient outcomes [3]. Common misdiagnoses include the following (Table 2):

- **Vascular lesions**: DFSP often presents with a reddish or purplish hue, which can easily lead clinicians to suspect vascular anomalies, such as hemangiomas or vascular malformations [29,30]. These benign conditions are common in infants, and DFSP similarity can result in inappropriate initial management or conservative follow-up, which delays proper treatment [29].
- **Benign proliferative lesions**: Conditions like hypertrophic scars, keloids, and fibromas are also frequently considered due to their appearance and benign nature [19,20,21]. These lesions are often characterized by localized skin thickening or growth, which may closely resemble DFSP, particularly in its plaque or nodular form. Misclassification as a benign proliferative lesion can lead to an underestimation of the potential seriousness of the condition, delaying the necessary surgical intervention [19,20,21,24,26].
- **Dermatofibromas and birthmarks**: Dermatofibromas are common benign fibrous lesions of the skin, and congenital DFSP may present in a similar manner, with slow-growing plaques or nodules. Birthmarks present from birth can also confuse the diagnosis, particularly when the lesions are not rapidly changing [30]. DFSP lesions present at birth are often assumed to be benign congenital nevi or vascular birthmarks, leading to diagnostic errors [3,30].
- **Other tumors**: Although rare, Giant Cell Fibroblastoma (GCF) can be mistaken for DFSP, as they share a close histogenetic relationship with GCF and DFSP, but differ in the degree of proliferative activity, which is much higher in the area of DFSP [26].
- **Atrophic-based skin conditions**: in the differential diagnosis of dermatofibrosarcoma protuberans (DFSP), atrophic-based skin conditions, some of which are rare, may be considered. These include mycosis fungoides anetodermica, granulomatous slack skin syndrome, mid-dermal elastolysis, nevus anelasticus, and the congenital atrophic variant of DFSP. The differential diagnosis is made at the histopathological level [31].

**Table 2:** Common misdiagnoses of congenital DFSP.

Category of Misdiagnosis	Reason for Misdiagnosis	Clinical Implications	Ref.
Vascular lesions: - Hemangiomas - Vascular malformations	Similar coloration (reddish, purplish, bluish), common in neonates and infants	Inappropriate initial management. Delayed diagnosis of malignant potential	[32,33]
Benign lesions: - Hypertrophic scars - Keloids	Similar appearance as firm, raised growths; benign nature often leads to underestimation of severity	Assumption of non-malignancy results in delayed treatment and potential lesion growth	[20,21]
Fibromas			
Dermatofibromas	Slow-growing, firm plaques resembling benign congenital skin lesions	Misinterpretation as a common benign lesion can prevent timely biopsy and histopathological confirmation	[19]
Pigmented lesions	Presence of pigmentation resembling other benign or even malignant pigmented lesions	Delayed accurate diagnosis due to misclassification as benign nevi or melanoma	[18,25]

### 5.2. Factors Contributing to Misdiagnosis

Several factors contribute to the frequent misdiagnosis of congenital DFSP:

- **Rarity of the condition**: Congenital DFSP is exceptionally rare, and its occurrence is something that many general practitioners, dermatologists, and pediatricians may never encounter during their careers. This lack of familiarity leads to an understandable but significant diagnostic gap. When faced with an unusual lesion, clinicians may be more inclined to diagnose more common, benign conditions rather than consider a rare sarcoma [3,15,16].
- **Variable clinical presentation**: Congenital DFSP has a wide range of appearances, including skin-colored, reddish, purplish, or bluish plaques or nodules. The variability of presentation, particularly when the lesion mimics other benign conditions, creates diagnostic confusion [19,20,21,22,23,24]. The slow growth pattern of DFSP also contrasts with what many clinicians associate with malignant lesions, adding to the diagnostic difficulty [34].
- **Anatomical complexity**: The location of congenital DFSP lesions plays an important role in diagnosis. Lesions located in the head and neck or lower limbs are particularly challenging due to the anatomical complexity and the number of benign entities that present similarly in these areas. For example, a lesion in the head and neck may be mistaken for a benign cyst or vascular malformation due to the wide range of benign masses typically seen in this region [3,19,22,23,26].
- **Overlapping symptoms**: Both benign and malignant lesions may exhibit slow growth, firm texture, and a lack of alarming symptoms such as pain or rapid change. The indolent nature of DFSP, with its characteristic slow but steady progression, often leads to a false sense of security among clinicians and parents alike, leading to delays in further investigation [3,34,35].

### 5.3. Importance of Early Diagnosis

The importance of early diagnosis in congenital DFSP cannot be overstated. When diagnosis is delayed, there are several negative outcomes that become more likely, including the following:

- **Increased tumor size**: Due to its indolent but steady growth, if not recognized early, congenital DFSP can grow significantly in size before proper treatment is initiated. As the lesion increases in size, it becomes more complex to treat, often involving deeper invasion into underlying tissues, including muscle and sometimes even bone [5,12].
- **More extensive surgery**: Early diagnosis allows for a more conservative approach to surgical excision. However, as the lesion grows larger, wider excision becomes necessary to ensure complete removal and prevent recurrence. The larger the surgical excision, the more tissue must be sacrificed, which can lead to greater functional limitations, increased morbidity, and a more noticeable cosmetic defect [5,6,12].
- **Higher risk of recurrence**: DFSP is locally aggressive, with a high tendency to recur if not entirely removed. The risk of recurrence increases significantly if the initial surgical excision is incomplete, which can occur more frequently when diagnosis is delayed. Early, precise surgical management, particularly using techniques like Mohs micrographic surgery, is key to minimizing recurrence [5,6,7].
- **Psychological impact**: The need for larger surgeries and the potential for recurrence have significant psychological consequences, particularly in pediatric patients. Visible scars and potential disfigurement can have a lasting impact on a child’s self-esteem and quality of life. This highlights the importance of early, precise intervention that minimizes scarring and preserves as much healthy tissue as possible [36].

## 6. Diagnostic Flowchart

**Figure 1:**
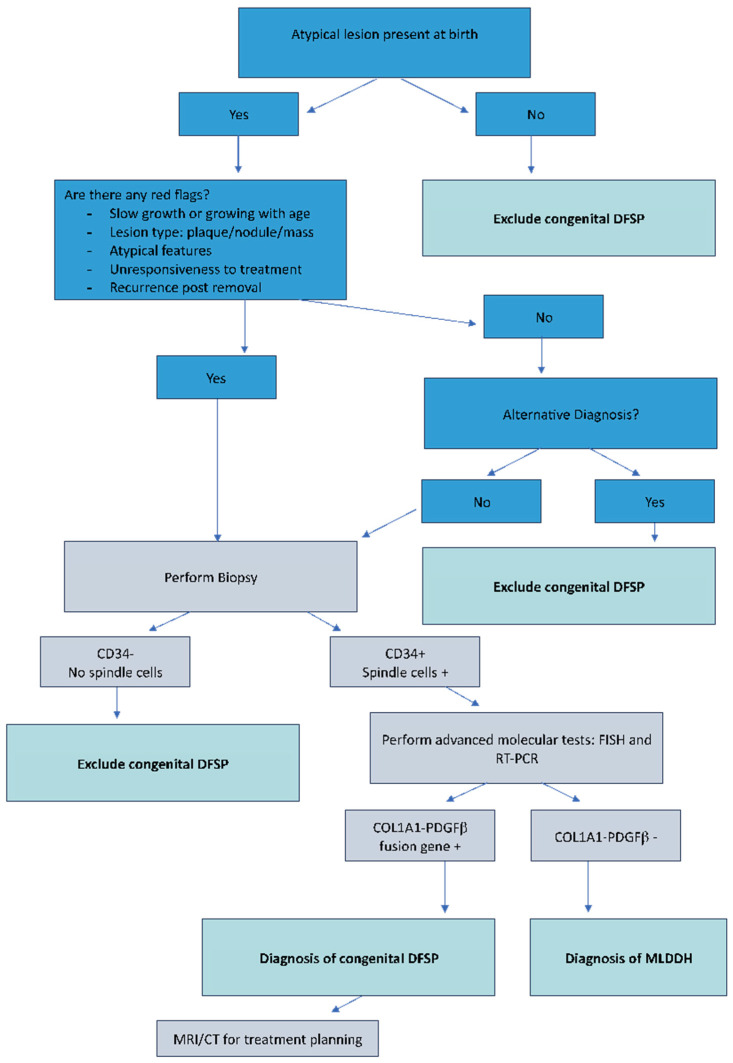
Operative flowchart to aid in the diagnosis of DFSP.

- **Clinical evaluation**: The diagnostic approach to congenital DFSP begins with a thorough clinical evaluation. A high index of suspicion is paramount, particularly when clinicians encounter congenital skin lesions that exhibit atypical features or fail to respond to conventional treatments. Congenital DFSP often mimics benign lesions, such as hemangiomas or dermatofibromas, making a cautious and investigative approach essential [29,32,33]. Clinicians should be alert to slow-growing, firm plaques or nodules, especially those that do not resolve or behave atypically over time [37,38]. A detailed patient history and examination are also critical components of clinical evaluation [19]. Assessing the growth rate, characteristics of the lesion (e.g., color, firmness, location), and noting any changes over time can help differentiate DFSP from more common benign conditions. The presence of a lesion at birth that slowly grows, remains persistent, or becomes more irregular should prompt further investigation [6,16,34]. (Figure 1)
- **Biopsy and histopathology**: An early biopsy is recommended for any congenital lesion that appears atypical or shows no response to initial treatments. A biopsy provides definitive information regarding the nature of the lesion [12]. Histopathologically, DFSP is characterized by spindle-shaped cells arranged in a storiform or cartwheel pattern, an important distinguishing feature [10]. Immunohistochemistry is also valuable; DFSP usually shows strong CD34 positivity, which serves as a useful diagnostic marker to differentiate it from other skin conditions [10].
- **Molecular testing**: Molecular testing plays a key role in confirming the diagnosis of DFSP, particularly in cases where clinical and histopathological findings are inconclusive. Detection of the COL1A1-PDGFB fusion gene, a result of the characteristic chromosomal translocation t(17;22)(q22;q13), is highly specific to DFSP and serves as a definitive diagnostic marker [8]. Advanced molecular techniques, such as FISH and RT-PCR, allow the precise identification of this genetic rearrangement and distinguish DFSP from its benign mimics, including MLDDH [8,9,10,11].
- **Imaging studies**: For a comprehensive assessment of the lesion, imaging studies like MRI and CT scans may be utilized, particularly when the lesion involves complex anatomical areas or deeper tissue layers. MRI provides detailed images that can help evaluate the extent of soft tissue involvement, while CT scans can be useful for assessing the depth of invasion and involvement of surrounding structures. These imaging modalities are essential for surgical planning, especially in cases where the lesion is extensive or involves critical areas such as the head, neck, or extremities. Imaging helps delineate the tumor margins, providing crucial information that guides the extent of surgical excision needed to achieve negative margins and minimize recurrence risk [12,13].
- **Emerging imaging technologies**: emerging imaging technologies hold significant promise for improving the diagnostic accuracy and management of DFSP, particularly when it comes to differentiating it from other cutaneous conditions. Among these, multispectral imaging has shown potential in analyzing the molecular and structural characteristics of skin lesions, providing enhanced visualization of subtle differences in tissue composition. This non-invasive technique could aid in early detection and more precise delineation of tumor margins, even in anatomically complex regions [39,40]. Similarly, optical coherence tomography (OCT) and photoacoustic imaging are gaining attention as advanced tools for real-time, high-resolution imaging of skin layers, enabling detailed assessment of lesion depth and vascular involvement [41,42]. While these technologies offer exciting possibilities, they remain largely experimental and require further validation through extensive clinical studies.

## 7. Differential Diagnosis Between Congenital DFSP and MLDDH

A particularly challenging differential diagnosis in congenital DFSP cases is MLDDH, a benign congenital lesion with clinical and histological features that closely mimic DFSP. MLDDH typically presents as an erythematous to yellow-brown atrophic plaque on the neck or trunk with overlapping features such as spindle cell proliferation and strong CD34 positivity [9]. However, MLDDH is distinguished by diffuse factor XIIIa positivity, which is absent in DFSP, suggesting a dermal dendritic cell origin. The absence of the COL1A1-PDGFB fusion gene, a hallmark of DFSP, serves as a definitive diagnostic criterion, confirmed by molecular techniques such as FISH or RT-PCR [8,9]. Despite their critical role, these molecular diagnostic tools are not universally used, particularly in peripheral or resource-limited centers, where histopathology and immunohistochemistry alone may be relied upon. Without a high index of clinical suspicion, such advanced testing may not be pursued, increasing the risk of misdiagnosing MLDDH as DFSP [8,9]. This may lead to overly aggressive surgical intervention for a condition that is benign and does not require such invasive treatment. To avoid such pitfalls, clinicians must recognize the importance of combining clinical judgment with comprehensive immunohistochemical and molecular evaluation to ensure accurate diagnosis and appropriate management.

## 8. Treatment

### 8.1. Surgical Management

- Wide local excision: this is a traditional method aiming to achieve clear margins to reduce recurrence risk [5,12].
- Mohs micrographic surgery: this treatment method is preferred in many cases due to its tissue-sparing benefits and higher cure rates [7].
- Advantages in pediatrics: surgical management preserves healthy tissue, reducing the functional and psychological impact of the treatment [36].

### 8.2. Adjuvant Therapies

- Radiation Therapy: this treatment is considered in cases where surgical margins are positive, or surgery is not feasible [36].
- Targeted molecular therapy involving imatinib, a tyrosine kinase inhibitor that can be effective against tumors expressing the PDGFB receptor [14].
- Indications: these therapies are recommended for unresectable, recurrent, or metastatic cases [43].

### 8.3. Multidisciplinary Approach

- Team involvement: dermatologists, pediatricians, oncologists, pathologists, and surgeons collaborate to provide optimal care [13].
- Individualized treatment plans: treatments should be tailored based on the patient’s age, lesion size, location, and the treatment’s potential impact on the patient’s growth and development [13].

### 8.4. Prognosis and Follow-Up

- Recurrence risk: the risk of recurrence is high if margins are not clear; hence, long-term follow-up is essential [5].
- Monitoring: regular clinical examinations and imaging should be performed when indicated [44].
- Psychosocial support: the emotional and psychological needs of pediatric patients and their families should be addressed [36].

## 9. Recommendations for Clinicians

Congenital DFSP is an uncommon but potentially serious condition that requires a high level of clinical awareness to ensure timely and effective treatment. Given the challenges associated with its diagnosis and management, we present the following recommendations which should help clinicians optimize patient outcomes [36].

- Maintain vigilance:

Clinicians should maintain a high index of suspicion for congenital DFSP in the differential diagnosis of congenital skin lesions with atypical features, particularly those that are persistent, slow-growing, or unresponsive to standard treatments [6,16,34,44]. Given its rarity and resemblance to more common benign conditions like vascular malformations, dermatofibromas, or congenital nevi, DFSP is often overlooked [3,19,30,37]. Clinicians should approach congenital lesions that exhibit irregular growth or unusual characteristics with caution, considering a broader differential that includes malignant possibilities [12].

- Early intervention:

Early intervention is critical to improving outcomes in congenital DFSP. A prompt biopsy is recommended for any congenital skin lesion that appears suspicious, does not respond to initial management, or demonstrates unusual growth [17,24]. A biopsy, followed by histopathological examination, can provide definitive information about the lesion, facilitating early treatment [10,12]. Early diagnosis not only ensures that the lesion is identified correctly but also limits the need for more extensive surgical procedures that may be required if the tumor is allowed to grow unchecked [7,19,29].

- Patient education:

Educating families is a crucial component of managing congenital DFSP. Families should be informed about the importance of early diagnosis, the need for further testing when skin lesions do not behave as expected, and the various treatment options available, including surgery [5,7,12]. Open communication with families helps ensure compliance with follow-up care, reduces anxiety, and allows parents to understand the rationale behind surgical intervention and the importance of monitoring for recurrence [29,36].

- Optimize surgical outcomes:

The treatment of congenital DFSP often involves surgery, and it is important to balance oncological control with minimizing morbidity, particularly in pediatric patients [5,7,12]. Optimizing surgical outcomes includes employing tissue-sparing techniques, such as Mohs micrographic surgery, when possible. This approach enables precise removal of the tumor while conserving as much healthy tissue as possible, which is particularly significant for growing children, in whom functional and aesthetic outcomes are a major concern [7]. When Mohs surgery is not available, wide local excision with histopathological margin assessment should be performed to reduce the risk of recurrence while considering the impact on the child’s development and quality of life [5,12].

## 10. Conclusions

In conclusion, misdiagnosis of skin lesions is a significant clinical issue that is frequently documented in the literature. Skin tumors can often mimic other dermatological conditions, making early and accurate diagnosis a critical challenge for physicians [45,46,47,48,49]. In this context, congenital DFSP represents a rare but significant challenge in pediatric dermatology due to its unusual presentation and its tendency to mimic benign skin conditions, such as vascular malformations, hemangiomas, or congenital nevi [15,16,18,32,33]. The rarity of congenital DFSP, combined with its typically subtle early presentation, often leads to misdiagnosis or delayed recognition [3,15,16]. As a result, many children with congenital DFSP experience treatment delays that can contribute to more extensive surgical requirements, an increased risk of recurrence, and greater overall morbidity [12,37]. Early recognition and accurate diagnosis are of paramount importance when it comes to managing congenital DFSP effectively [37]. Unlike more common benign lesions, which are typically managed conservatively, DFSP requires definitive surgical intervention [7,12]. The indolent but infiltrative nature of DFSP can result in considerable tissue invasion if not treated promptly. Early diagnosis allows for smaller, more tissue-sparing surgical procedures, significantly reducing the risk of long-term functional and cosmetic impairment in pediatric patients [7,11,12,19]. Wide local excision, ideally performed with Mohs micrographic surgery to ensure complete removal while minimizing healthy tissue loss, remains the cornerstone of treatment [5,6,7,12]. The role of the clinician is pivotal in ensuring early detection. General practitioners, pediatricians, and dermatologists should maintain a high index of suspicion when evaluating congenital skin lesions that present atypical features or fail to respond as expected to standard therapies [6,16,34,44]. Any lesion that continues to grow, fails to regress, or presents with features such as firmness, irregular growth, or discoloration warrants closer evaluation and an early biopsy. Awareness of congenital DFSP among healthcare providers can lead to timely histopathological examination, allowing for definitive diagnosis and appropriate management [10,12]. A multidisciplinary approach is essential for managing congenital DFSP. The rarity and complexity of the condition necessitate collaboration across multiple specialties, including dermatology, pediatric surgery, pathology, and oncology. Dermatologists play a crucial role in identifying suspicious lesions, while pediatric surgeons are instrumental in executing precise, tissue-sparing excisions that limit disfigurement and functional impairment [13]. Pathologists confirm the diagnosis via histopathological examination and immunohistochemistry, while oncologists may be involved in cases where advanced or recurrent disease requires targeted therapies. A collaborative effort of this nature ensures that every aspect of the patient’s care is optimized to yield the best possible outcome [10,12,50]. In addition to medical and surgical management, the psychological and developmental impact of congenital DFSP should not be overlooked [36]. Surgical excision, particularly of large or prominent lesions, can have significant implications for the child’s physical appearance and psychological well-being [12]. Therefore, providing psychosocial support to the patient and their family is vital, as this will alleviate anxiety and foster a positive outlook toward treatment and recovery. Parents must be educated about the importance of early detection, treatment options, and long-term follow-up to manage expectations and encourage adherence to medical advice [29,36]. Finally, long-term follow-up is necessary to ensure that patients do not experience local recurrence, which can occur even after seemingly complete excision. Regular clinical assessments are essential for monitoring the treatment site and ensuring prompt intervention should a recurrence be detected [43]. With heightened clinical awareness, proactive diagnostic strategies, and a well-coordinated multidisciplinary approach, the prognosis for patients with congenital DFSP can be significantly improved [13]. Early intervention not only reduces the need for more aggressive treatments but also minimizes the potential for long-term morbidity, thereby enhancing the quality of life of affected children and their families [7,19,29,51]. By following these recommendations and focusing on early detection and comprehensive management, clinicians can help overcome the diagnostic challenges presented by congenital DFSP and contribute to better health outcomes for this vulnerable population [19,29]. To conclude, we present an operational flow chart for pediatricians and dermatologists to aid in the diagnosis of DFSP and to prevent potential diagnostic errors or delays (Figure 1).

## Figures and Tables

**Table 1:** Clinical characteristics of congenital DFSP.

Lesion Type	Description	Differential Diagnoses	Ref.
Plaques	Flat or slightly raised, firm areas	Dermatofibromas, keloids, hypertrophic scars	[19,20,21]
Nodules	Firm, well-circumscribed masses	Lipomas, cysts, neurofibromas	[22,23,24]
Masses	Larger, more prominent growths	Sarcomas, deep-seated lipomas	[14,22,23]
Pigmented Lesions	Darker-colored lesions resembling melanocytic nevi or melanoma	Pigmented nevi, melanoma	[18,25]
